# Maladaptive changes in the homeostasis of AEA-TRPV1/CB1R induces pain-related hyperactivity of nociceptors after spinal cord injury

**DOI:** 10.1186/s13578-025-01345-6

**Published:** 2025-01-09

**Authors:** JiaQi Hu, WenYong Fan, Yue Xu, XiaoFei Li, HaoYang Zhang, Shun Li, Lei Xue

**Affiliations:** 1 State Key Laboratory of Medical Neurobiology and MOE Frontiers Center for Brain Science, Fudan University, Shanghai, 200438 People’s Republic of China; 2 Department of Physiology and Neurobiology, School of Life Sciences, Fudan University, Shanghai, 200438 People’s Republic of China; 3 Center for Rehabilitation Medicine, Department of Pain Management, Zhejiang Provincial People’s Hospital, Affiliated People’s Hospital, Hangzhou Medical College, Hangzhou, 310014 People’s Republic of China; 4 Key Laboratory of Spine and Spinal Cord Injury Repair and Regeneration, Ministry of Education, Department of Orthopedics, Tongji Hospital, School of Medicine, Tongji University, Shanghai, 200092 People’s Republic of China; 5 Research Institute of Intelligent Complex Systems, Fudan University, Shanghai, 200433 People’s Republic of China

**Keywords:** Spinal cord injury, Anandamide, DRG, Action potentials, Hyperexcitability

## Abstract

**Background:**

Neuropathic pain resulting from spinal cord injury (SCI) is associated with persistent hyperactivity of primary nociceptors. Anandamide (AEA) has been reported to modulate neuronal excitability and synaptic transmission through activation of cannabinoid type-1 receptors (CB1Rs) and transient receptor potential vanilloid 1 (TRPV1). However, the role of AEA and these receptors in the hyperactivity of nociceptors after SCI remains unclear.

**Results:**

In this study, we investigated the effects of AEA and its receptors on the hyperexcitability of mouse dorsal root ganglion (DRG) neurons after SCI. Using a whole-cell patch-clamp technique, we found that the timing of SCI-induced hyperexcitability in nociceptors paralleled an increase in the endocannabinoid AEA content. The expression of TRPV1 and CB1R was also upregulated at different time points after SCI. High-dose extracellular administration of AEA increased the excitability of naive DRG neurons, leading to the transition from a rapidly accommodating (RA) hypoexcitable state to a highly excitable non-accommodating (NA) state. These AEA-induced transitions were facilitated by increased TRPV1 transcription. Pharmacological and Ca^2+^ imaging experiments revealed that AEA induced hyperexcitability in nociceptors after SCI via the AEA-TRPV1-Ca^2+^ pathway, whereas activation of CB1Rs reduced SCI-induced hyperexcitability and maintained cytosolic Ca^2+^ concentration ([Ca^2+^]_cyto_) at low levels in the early stages of SCI. As the AEA and TRPV1 levels increased after SCI, adaptive neuroprotection transitioned to a maladaptive hyperactive state, leading to sustained pain.

**Conclusions:**

Taken together, this study provides new insights into how endocannabinoids regulate nociceptor activity after SCI, offering potential targets for the treatment of neuropathic pain.

**Supplementary Information:**

The online version contains supplementary material available at 10.1186/s13578-025-01345-6.

## Background

SCI is one of the most challenging traumatic diseases, leading to damage in both central and peripheral nerves [1]. Nearly half of SCI patients suffer from chronic neuropathic pain for the rest of their lives [2]. Existing treatments aimed at reducing pain have limited effectiveness, partly because the mechanisms that lead to the onset and progression of pain are not fully understood [3]. The hyper-excitation of nociceptors is well-documented as a key factor in neuropathic pain [4]. Following spinal contusion at the thoracic level, nociceptors below the injury exhibit hyperexcitability and spontaneous action potentials (sAPs) for months, correlating with behavioral indicators of pain [5]. Elucidating the mechanisms that promote nociceptor hyperactivity may reveal promising targets for treating neuropathic pain after SCI.

Numerous extrinsic injury-related signals, such as lipopolysaccharide (LPS), pro-inflammatory cytokines, and adenosine triphosphate (ATP), can directly stimulate and sensitize nociceptors [6–9]. However, the role of these signals in promoting nociceptor hyperactivity after SCI is not well understood. According to the hypothesis proposed by Edgar T. Walters, certain signals may be continuously released from the time of injury until the onset of neuropathic pain, causing nociceptors to enter a maladaptive hyperactive state [10]. This results in hyperalgesia and long-term spontaneous pain that develops several months after the injury [11]. Anandamide (N-arachidonoyl-ethanolamine, AEA), an endocannabinoid, acts as an inhibitory retrograde neuromodulator in the central nervous system and provides a neuroprotective effect via the CB1R [12, 13]. Activation of CB1R can inhibit calcium influx by modulating presynaptic voltage-gated calcium channels (VGCCs), inhibiting synaptic transmission [14]. Therefore, the AEA-CB1R signaling pathway is often considered an inhibitory signal in neurotransmission [15]. However, the effect of AEA on pain-related hyperactivity in nociceptors is complex and controversial. For example, AEA levels in the DRG increase following spinal nerve ligation (SNL)-induced neuropathic pain, which is thought to be a homeostatic response to reduce pain [16]. In line with this, reduced AEA levels contribute to pain maintenance in a mouse model of bone cancer [17]. However, an increase in AEA has been suggested to trigger visceral hyperalgesia by altering receptor expression and sensitivity [18].

The dual effect of AEA in sensory afferent modulation is thought to target different endogenous receptors [19]. The effect of AEA could be attributed to its activation of TRPV1, as well as CB1R [20]. TRPV1 is a non-selective cation channel found in sensory neurons [21]. Ions, especially Ca^2+^, that permeate through TRPV1 channels lead to downstream signaling activation, which further triggers inflammation and facilitates the transmission of pain-related signals [22]. Some evidence suggests that SCI increases the expression of TRPV1 and enhances the sensitivity of isolated nociceptors to TRPV1 agonist capsaicin [23]. However, the potential connection between AEA, its receptors, and the increased activity of nociceptors caused by SCI has not been explored.

In this study, we demonstrate that the increase in AEA following SCI induces hyperexcitability in nociceptors via the AEA-TRPV1 signaling pathway. Furthermore, levels of the neuroprotective receptor CB1R increase in the early stages of SCI. The AEA-CB1R signaling may provide an adaptive neuroprotective effect at the onset of the injury. Due to the upregulation of both AEA and TRPV1, nociceptors transit into a maladaptive state of hyperexcitability and hypersensitivity, contributing to chronic neuropathic pain induced by SCI. These findings may enhance our understanding of how endocannabinoids regulate nociceptor hyperactivity after SCI.

## Methods

### Animals and injury procedures

Female C57BL/6 mice weighing approximately 20 g and aged between 8 to 10 weeks were maintained in a controlled environment with a 12-h light/dark cycle at a temperature of 22 ± 1 °C. Standard food and water were provided regularly.

SCI was induced as described previously [24, 25]. All animals were anesthetized with inhalant isoflurane (2%) delivered in oxygen-enriched air using a dissecting microscope (Stemi 508, Carl Zeiss, GER) and rodent stereotaxic apparatus (68,037, RWD, CN). First, laminectomy was performed at T8-T10 to expose the spinal cord. Next, SCI was induced at the T9 level by administering a moderate contusion injury (60 kilodynes) using a spinal cord impactor (MASCIS model III, W. M. Keck, USA). For the sham group, only the laminectomy was performed, without subsequent crush injury. Finally, the animals were monitored daily for infection, abnormal wound healing, or weight loss. The mouse’s bladder was manually emptied twice a day until euthanasia. All procedures were approved by the Animal Care and Use Committee of Fudan University and followed the guidelines of the International Association for the Study of Pain.

### Dissociation and culture of DRG neurons

DRGs were dissociated using previously described methods [26, 27]. Both sides of the L1-L6 DRGs were isolated in ice-cold Hank’s Balance Salt Solution (HBSS, Gibco, USA) bubbled with 5% CO_2_ and 95% O_2_. The tissues were digested with collagenase type I (0.2 mg/mL, Sigma, USA) and dispase II (3 mg/mL, Sigma, USA) at 37℃ for 45 min each. After digestion, tissues were dissociated with a Pasteur pipette and then seeded on poly-D-lysine-coated (0.1 mg/mL, Sigma) coverslips. Neurons were cultured in DMEM/F12 (Gibco, US) supplemented with 10% fetal bovine serum (Gibco, US). For electrophysiological study, cells were used within 12 h after seeding.

### Measurement of AEA by LC–MS/MS

DRG samples used for liquid chromatography-mass spectrometry (LC–MS) analysis were prepared using the previously reported method [16, 28]. Briefly, DRGs and spinal cord segments at the L1-L6 level, from both the sham and SCI groups, were homogenized and added to internal standards (AEA-d4, Sigma, USA) containing 2.0 mL of methanol. The supernatants were then extracted with chloroform and centrifuged at 3000 rpm for 10 min at 4 °C. The organic phases were dried under a stream of nitrogen, and the residues were re-dissolved in 2 mL of methanol. All samples were analyzed by mass spectrometry (QTrap 6500, SCIEX, UK) through selected reaction monitoring. For quantitative analysis, the peak area of anandamide ions from the test samples was compared and normalized.

### Western blot analysis

L1-L6 DRGs were homogenized and extracted using RIPA lysis buffer (Beyotime, CN) mixed with a protease inhibitor cocktail (Selleckchem, USA). Samples were centrifuged at 12,000 rpm for 10 min at 4℃. The supernatant was separated, and its protein concentration measured using the bicinchoninic acid (BCA) method (Bio-Rad, USA). Based on the protein concentration, samples were diluted with lysis buffer, and 30 μg of protein was loaded into each well. After electrophoresis, the gel was transferred to polyvinylidene fluoride (PVDF) membranes (Merck, USA), which were then blocked with 5% fat-free milk in phosphate buffered saline (PBS) solution with Tween-20. The membrane was incubated overnight at 4℃ with an antibody against TRPV1 (anti-rabbit, 1:400, Alomone, IL), CB1R (anti-rabbit, 1:100, Abcam, UK), or β-actin (anti-mouse, 1:1000, Santa Cruz Biotechnology, USA). After being washed with a PBS solution containing Tween-20, the membrane was incubated at room temperature for 1 h with goat anti-mouse (1:5000, CST, USA) or goat anti-rabbit (1:5000, CST, USA). Protein expression was quantified using enhanced chemiluminescence (ECL) reagent horseradish peroxidase (HRP)-linked secondary antibodies (Thermofisher, USA) and by determining the density of the target band in Image J software (v1.8.0, NIH, USA).

### Immunofluorescence

DRG neurons (L4-L5) were fixed in 4% paraformaldehyde (PFA) for 12 h and then dehydrated in a 30% sucrose solution for over 2 nights at 4 °C for 36–48 h. Longitudinal sections of DRG (14 μm) were made using a cryostat (CM1950, Leica, GER), and then incubated with primary antibodies for TRPV1 (1:100, Alomone, IL) and CB1R (1:100, Alomone, IL) in combination with antibodies for neurofilament-200 (NF200, 1:500, Abcam, UK), calcitonin gene-related peptide (CGRP, 1:200, Abcam, UK), and isolectin B4 (IB4, 1:100, Sigma, USA). After washing, the sections were incubated with Cy3 or 488 secondary antibodies (1:500, Beyotime, CN) for 30 min at room temperature (22–24 ℃). All images were captured under identical parameters by a confocal microscope (LSM700, Carl Zeiss, GER). The immunofluorescent staining was quantified using ImageJ software (v1.8.0, NIH, USA).

### Electrophysiology

Electrophysiological recordings were performed as described previously [29, 30]. All experiments were performed at room temperature (22–24 ℃). To record the action potential (AP), we used an extracellular solution (300–310 mOsm) with a pH of 7.4 containing the following (in mM): 125 NaCl, 2.5 KCl, 1 MgCl_2_, 2 CaCl_2_, 25 NaHCO_3_, 1.25 NaH_2_PO_4_, 25 glucose, 0.4 ascorbic acid, 3 myo-inositol, 2 sodium pyruvate. The intracellular solution (290–300 mOsm) contained (in mM): 125 K-gluconate, 20 KCl, 4 MgATP, 10 Na2-phosphocreatine, 0.3 Na_3_GTP, 10 HEPES, and 0.5 EGTA at a pH of 7.2. Pipette resistance was 3–6 MΩ, and only small DRG neurons with a soma diameter ≤ 30 μm were selected for recording. sAPs were recorded at the current clamp mode, holding at 0 pA for 3 min. If sAP was not detected and the resting membrane potential was < − 40 mV, the evoked AP (eAP) threshold and spikes were recorded further. The AP threshold was evoked using a series of 5-ms depolarizing current injections in 10 pA steps from − 10 pA. The current that induced the first action potential was defined as 1 × rheobase. NA and RA neurons were identified by stimulating neurons with a 2-s step protocol of 2 × rheobase injection currents to induce AP spike responses. If only a single AP was induced, the neuron was classified as RA. If repetitive AP was observed, the neuron was classified as NA [30]. The custom program for analyzing depolarizing spontaneous fluctuations (DSFs) was written in Python (v3.8.7), which allows us to quantify the irregular curves observed in our recordings. In this study, the minimum amplitude and duration of DSF were defined as 1.5 mV and 10 ms, consistent with a previous study [30, 31].

### Single-cell RT-qPCR

After electrophysiological recordings, we amplified the total RNA of single DRGs using a Single-Cell Sequence-Specific Amplification Kit (Vazyme, CN). We used ChamQ Universal SYBR qPCR Master Mix (Bio-Rad, USA) for RT-PCR analysis and analyzed the results with CFX manager software (Bio-Rad, USA). The primers to amplify the target genes are listed in Table 1. All measurements were made three times for each experiment. The β3-tubulin mRNA level was used as an internal reference. Cells with a Ct threshold ≥ 24 (adjusted for primer efficiency and dilution) were not included in the analysis [32]. Pre-amplified cells with Cq values > 35 were defined as not expressing [33].

**Table 1:** Primer sequences for RT-PCR

Gene name	Oligo primers (5′-3′)	
*Trpv1*	Forward:	TTGTGGAGGTGGCAGATA
	Reverse:	TCTTGTTGGTGAGTTCTTCT
*Trpv2*	Forward:	TGACTCGGCATACACAGA
	Reverse:	AACATTCGCTCCATTCTCTA
*Trpv3*	Forward:	TAACCAGCCTGAGATTGTG
	Reverse:	AACGAAGTCATTCTGAGTCT
*Trpv4*	Forward:	GTCTCGCAAGTTCAAGGA
	Reverse:	TCTACAGCCAGCATCTCA
*Trpm8*	Forward:	TCTTCTACATCGCCTTCCT
	Reverse:	CCACTGCCTCACTTCATC
*Cnr1*	Forward:	TGCTGTTGCTGTTCATTG
	Reverse:	CACCTTGCCATCTTCTGA
*Cnr2*	Forward:	CTTCGCCTTCTGTTCCAT
	Reverse:	ATACTTCTTCCAGCCTATCAG
*Tubb3*	Forward:	CCAGCGGCAACTATGTAG
	Reverse:	GGTTCCAGGTTCCAAGTC

### Calcium imaging

For intracellular calcium imaging, DRG neurons were rinsed twice with HBSS (Gibco, USA), which contains calcium and magnesium, to remove the culture medium. The cells were incubated with Fura-2AM (Thermo Fisher Scientific, USA), at a final concentration of 5 μM (dissolved in HBSS), for 40 min at 37℃. After loading the neurons with Fura-2AM, the cultures were washed three times with the extracellular solution (the same solution used for electrophysiology) and incubated for an additional 10 min. The neurons were then moved to the imaging set-up. Fura-2AM signals were captured using a microscope (Eclipse Ti, Nikon, JPN) equipped with a sCMOS camera (ORCA-Flash4.0, Hamamatsu, JPN). The 340/380 light sources were generated by a Lambda DG4 Plus illumination system (Sutter, USA). The cell somas were identified as regions of interest (ROIs) using Metafluor software (Molecular Devices, USA). The basal [Ca^2+^]_cyto_ was recorded for 120 s and used to normalize the ratio of F340/380 in different groups before AEA administration.

### Drug administration

TRPV1 antagonist capsazepine (Alomone, IL), CB1R agonist WIN55,212–2 (Sigma, USA), and CB1R antagonist AM-251 (Sigma, USA) were prepared as stock solutions in DMSO (< 0.1%, Sigma, USA). These solutions were stored as aliquots at -20 °C.

### Statistical analysis

Electrophysiological recordings were made using Igor Pro (v9.0.1, WaveMetrics, USA). The results were analyzed in SPSS Statistics (v20.0, IBM, USA) and Prism GraphPad software (v8.01, GraphPad Software Inc, USA). All data were presented as either mean ± SEM or incidence (% of neurons sampled). The normality of the datasets was evaluated using the Shapiro–Wilk test. Normally distributed data were assessed by unpaired Student’s t-test or one-way ANOVA with Dunnett’s post hoc test. Non-normally distributed data were analyzed using the Kruskal–Wallis one-way ANOVA with Dunn’s multiple comparison test. Incidence comparisons were made using Fisher’s exact test with Bonferroni corrections for multiple comparisons. A p value < 0.05 was considered significant.

## Results

### SCI induces increased AEA content and hyperactivity in nociceptors

**Fig. 1:**
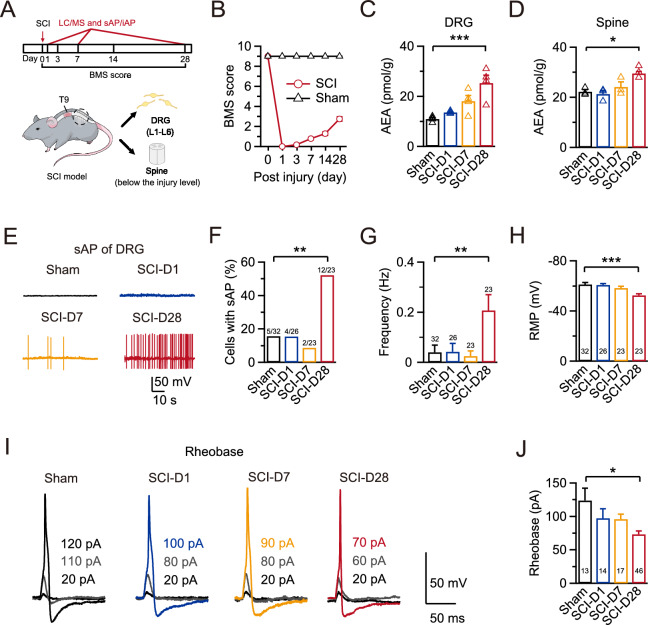
SCI induces increased AEA content and hyperactivity in nociceptors. **A** Schematic of the experimental design (upper) and anatomical positioning of SCI (lower), including monitoring mouse behavior, assessing AEA content, and the electrophysiological recordings at various time points post-SCI. **B** The BMS demonstrated hindlimb motor function recovery in the SCI (red) and sham (black) groups from 0 (D0) to 28 days (D28) after SCI. **C**, **D** Statistics for AEA content from different groups in DRGs (n = 4 for each group) and the spine below the injury level (n = 3 for each group). Comparisons among all four groups were performed using a one-way ANOVA with Dunnett’s post hoc test. *p < 0.05, ***p < 0.001. **E** Representative sAP recordings in DRGs using a whole-cell current-clamp configuration in the sham (black), SCI-D1 (blue), SCI-D7 (yellow), and SCI-D28 (red) groups. **F**–**H** Statistics for the sAP probability, firing frequency, and RMP in all four groups (Sham, n = 32, black; SCI-D1, n = 26, blue; SCI-D7, n = 23, yellow; SCI-D28, n = 23, red). A Fisher’s exact test was used for comparisons in F, whereas a Kruskal–Wallis one-way ANOVA with Dunn’s multiple comparison test was performed in **G** and **H**. **p < 0.01, ***p < 0.001. **I** Representative eAPs and their rheobase traces induced by depolarizing current injections in the sham (black), SCI-D1 (blue), SCI-D7 (yellow), and SCI-D28 (red) groups. **J** Statistics for the rheobase in all four groups (Sham, n = 13, black; SCI-D1, n = 14, blue; SCI-D7, n = 17, yellow; SCI-D28, n = 46, red) using a Kruskal–Wallis one-way ANOVA with Dunn’s multiple comparison test. *p < 0.05

Many studies have shown that increased AEA levels may cause hyper-excitation in nociceptors, leading to sustained pain. To examine the changes in AEA content and nociceptor excitability after SCI, we employed an experimental paradigm that included monitoring mouse behavior, assessing AEA content, and making electrophysiological recordings at various time points post-injury (Fig. 1A). Initially, the mice involved in this study had a score of 0 for both hind limbs on the Basso Mouse Scale (BMS) [34] one day after the SCI surgery. The mean scores remained below 3 from 1 to 28 days after the surgery (Fig. 1B). Next, we examined the changes in AEA content in DRGs and the spine below the injury level after SCI using LC–MS/MS. We quantified the level of AEA in DRG extracts using chromatography with AEA-d4 as an internal standard. In the sham group, the average AEA content in L1-L6 DRGs was 10.95 ± 0.54 pmol/g (n = 4; Fig. 1C). In contrast, the AEA content remained unchanged 1 to 7 days post-SCI. However, we observed a significant increase in AEA content 28 days post-SCI (25.32 ± 3.11 pmol/g, n = 4; p < 0.001; Fig. 1C). Similarly, AEA content in the spine below the injury level also increased 28 days post-SCI (Sham: 22.23 ± 0.84 pmol/g, n = 3; SCI day 28: 30.19 ± 1.34 pmol/g, n = 3, p = 0.012; Fig. 1D).

Having shown that the AEA content was significantly increased 28 days post-SCI, we evaluated the neurophysiological activities of DRGs following SCI by recording the sAPs in acutely dissociated DRG neurons with small diameters (≤ 30 μm) at various post-injury time points. The recordings were made in the whole-cell current-clamp configuration. In the sham group, sAPs were observed in only 5 out of 32 neurons (15.6%; Fig. 1E, upper left, F). However, 12 out of 23 neurons exhibited sAPs on 28 days post-SCI (52.2%; p = 0.007; Fig. 1E, lower right, F). The sAP frequency in DRG neurons significantly increased compared to the sham group 28 days post-SCI (Sham: 0.04 ± 0.03 Hz, n = 32; SCI: 0.21 ± 0.06 Hz, n = 23; p = 0.002; Fig. 1E, G). In addition, we observed significant depolarization of resting membrane potential (RMP) 28 days post-SCI (Sham: -60.2 ± 1.5 mV, n = 32; SCI: -51.4 ± 1.3 mV, n = 23; p < 0.001; Fig. 1H). However, no significant alterations were observed 1 and 7 days post-SCI in terms of sAP probability (Fig. 1G), or RMP (Fig. 1H) compared to the sham group. We also measured changes in the rheobase among the sham group and three time points post-SCI. Our results showed that rheobase was decreased only 28 days post-SCI compared to the sham group (Sham: 123.8 ± 18.5 pA, n = 13; SCI day 28: 73.0 ± 5.1 pA, n = 46; p = 0.037; Fig. 1I). These results suggest that the timing of SCI-induced hyperexcitability in nociceptors is paralleled by an increase in AEA content.

### TRPV1 and CB1R are upregulated in nociceptors following SCI

**Fig. 2:**
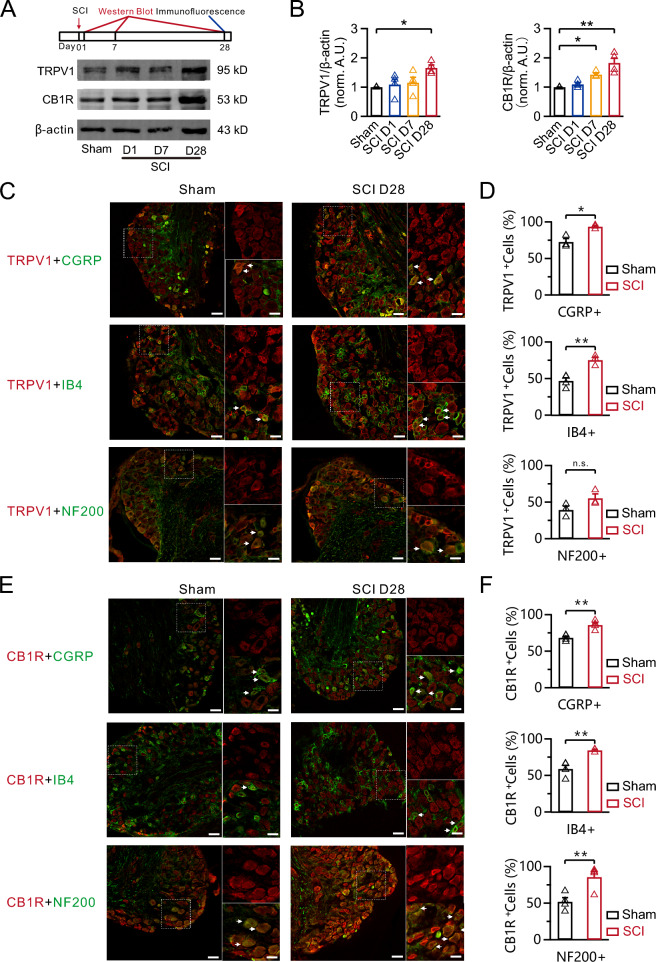
TRPV1 and CB1R are upregulated in nociceptors following SCI. **A** Schematic of the experimental design (top) and representative Western blots (bottom) showing the TRPV1 and CB1R levels in DRGs from the sham group or at different time points after SCI. **B** Statistics for the TRPV1 and CB1R levels from (**A**) using a one-way ANOVA with Dunnett’s post hoc test. *p < 0.05, **p < 0.01. **C** Immunofluorescence of TRPV1 (red) with CGRP (green, top), IB4 (green, middle), and NF200 (green, below) in DRG neurons in the sham and 28 days post-SCI groups. Scale bar, 50 μm. Scale bar of the enlarged view, 12.5 μm. **D** Statistics of co-localization of TRPV1 with CGRP, IB4, and NF200 in DRG neurons in the sham and 28 days post-SCI groups using a Fisher’s exact test. *p < 0.05, **p < 0.01. n.s., not significant. **E** Immunofluorescence of CB1R (red) with CGRP (green, top), IB4 (green, middle), and NF200 (green, below) in DRG neurons in the sham and 28 days post-SCI groups. Scale bar, 50 μm. Scale bar of the enlarged view, 12.5 μm. **F** Statistics of co-localization of CB1R with CGRP, IB4, and NF200 in DRG neurons in the sham and 28 days post-SCI groups using a Fisher’s exact test. **p < 0.01

AEA regulates neuronal excitability via activation of its receptors TRPV1 and CB1R. To investigate how the sensitivity of pain receptors is altered after SCI, we further examined the expression of TRPV1 and CB1R in DRG neurons following SCI using Western blot analysis. TRPV1 protein levels were significantly higher 28 days post-SCI compared to the sham group (Sham: 1.0, n = 4; SCI day 28: 1.7 ± 0.1, n = 4; p = 0.016; Fig. 2A, B, left). However, the levels remained unchanged in DRG neurons 1 and 7 days post-SCI (Fig. 2A, B, left). These findings are consistent with a previous report showing increased TRPV1 levels post-SCI [23]. Interestingly, changes in CB1R occurred earlier than changes in TRPV1. We observed an increase in CB1R levels 7 and 28 days after SCI, but no change in CB1R expression was observed in DRG neurons 1 day after SCI (Sham: 1.0, n = 4; SCI day 7: 1.4 ± 0.04, n = 4, p = 0.018; SCI day 28: 1.8 ± 0.2, n = 4, p = 0.001; Fig. 2A, B, right).

To identify specific cell types with increased TRPV1 following SCI, we used immunohistochemistry to examine the co-localization with CGRP, IB4, and NF200, which are markers of peptidergic C-type, nonpeptidergic C-type, and A-type neurons, respectively. Compared to the sham group (CGRP: 72.4 ± 4.7%, n = 3; IB4: 46.9 ± 4.2%, n = 4; NF200: 39.3 ± 5.2%, n = 3; Fig. 2C, D), the number of neurons expressing TRPV1 significantly increased in chronic nociceptor markers labeled with IB4 and CGRP, but not in NF200-labeled DRG neurons, 28 days post-SCI (CGRP: 93.5 ± 1.6%, n = 3, p = 0.014; IB4: 75.5 ± 4.1%, n = 3, p = 0.005; NF200: 55.2 ± 5.9%, n = 3, p = 0.116; Fig. 2C, D). Compared to the sham group (CGRP: 68.4 ± 2.2%, n = 4; IB4: 59.1 ± 5.1%, n = 4; NF200: 51.8 ± 5.8%, n = 4; Fig. 2E, F), the proportions of all three neuron types expressing CB1R significantly increased in DRG neurons 28 days post-SCI (CGRP: 85.7 ± 3.0%, n = 4, p = 0.004; IB4: 84.6 ± 1.4%, n = 3, p = 0.009; NF200: 87.9 ± 5.6%, n = 4, p = 0.004; Fig. 2E, F). These results suggest that the expression of TRPV1 and CB1R increases over time after SCI. More specifically, TRPV1 is primarily increased in neurons associated with chronic pain.

### High-dose AEA application on naive DRG can mimic the effects of SCI on nociceptors

**Fig. 3:**
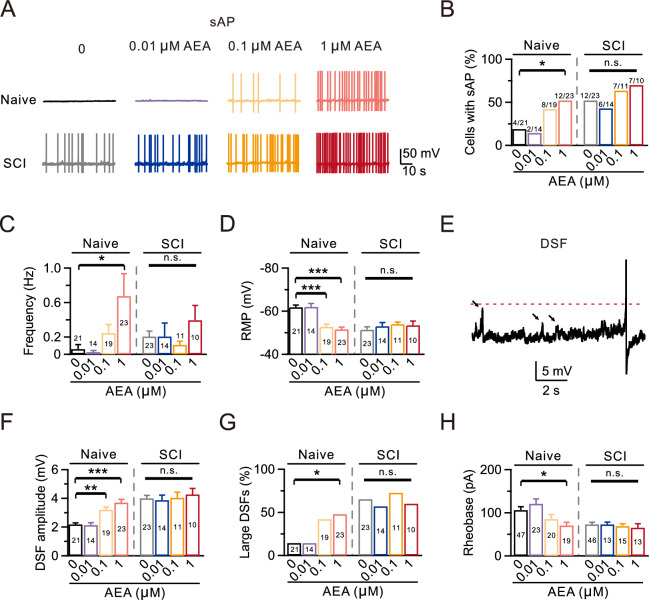
High-dose AEA can mimic the effects of SCI on nociceptors. **A** Top, representative sAP recordings using a whole-cell current-clamp configuration in the absence or presence of AEA in four naive groups. Bottom, similar to Top, but in four SCI groups. Different concentrations of AEA (0.01, 0.1, and 1 µM) were added to the extracellular solution at least 30 min before recordings were taken. **B**–**D** Statistics for the sAP probability, firing frequency, and RMP from various treatment groups (Naive, n = 21; Naive + 0.01 µM AEA, n = 14; Naive + 0.1 µM AEA, n = 19; Naive + 1 µM AEA, n = 23; SCI, n = 23; SCI + 0.01 µM AEA, n = 14; SCI + 0.1 µM AEA, n = 11; SCI + 1 µM AEA, n = 10). Comparisons between each dose and vehicle (0 µM) were made using a Fisher’s exact test in B, and a Kruskal–Wallis one-way ANOVA with Dunn’s multiple comparison test or a one-way ANOVA with Dunnett’s post hoc test in C and D. *p < 0.05, ***p < 0.001. n.s., not significant. **E** Representative DSFs after SCI (black arrowheads). **F**–**G** Statistics for the DSF amplitudes (**F**) and the incidence of large DSFs (**G**) in different treatment groups. Comparisons between each dose and vehicle (0 µM) were conducted using a Kruskal–Wallis one-way ANOVA with Dunn’s multiple comparison test (**F**) and a Fisher’s exact test (**G**). *p < 0.05, **p < 0.01, ***p < 0.001. n.s., not significant. **H** Statistics for the rheobase from all groups (Naive, n = 47; Naive + 0.01 µM AEA, n = 23; Naive + 0.1 µM AEA, n = 20; Naive + 1 µM AEA, n = 19; SCI, n = 46; SCI + 0.01 µM AEA, n = 13; SCI + 0.1 µM AEA, n = 15; SCI + 1 µM AEA, n = 13). Comparisons between each dose and vehicle (0 µM) were made using a Kruskal–Wallis one-way ANOVA with Dunn’s multiple comparison test. *p < 0.05. n.s., not significant

To clarify the specific effect of increased AEA levels on nociceptors post-SCI, DRG neurons isolated from both adult naive (adult mice that have not undergone any surgical procedures or interventions) and SCI mice at 28 days post-injury were exposed to AEA for 30 min. The AEA doses ranged from 0.01 to 1 μM, which closely match the reported ranges in acute and chronic rat spinal cords post-SCI (0.05–0.4 μM) [35, 36]. The sAPs were recorded under the current clamp configuration. In the naive group, sAP was observed in only 4 out of 21 DRG neurons (19.0%; Fig. 3A, B), whereas 12 out of 23 cells exhibited sAP in the presence of 1 μM AEA (52.2%; p = 0.031; Fig. 3A, B). No significant alterations in the probability of sAP were observed in the presence of 0.01 μM or 0.1 μM AEA (Fig. 3A, B). We also observed a significant increase in the sAP frequency in the presence of 1 μM AEA compared to the naive group, but not with 0.01 μM or 0.1 μM AEA (Naive: 0.06 ± 0.05 Hz, n = 21; Naive + 1 μM AEA: 0.68 ± 0.26 Hz, n = 23, p = 0.027; Fig. 3A, C).

We also investigated the effect of AEA on RMP. RMP was elevated in both the 0.1 μM AEA and 1 μM AEA groups compared to the naive group (Naive: -61.8 ± 1.0 mV, n = 21; 0.1 μM AEA: -52.8 ± 1.2 mV, n = 19, p < 0.001; Naive + 1 μM AEA: -51.6 ± 1.0 mV, n = 23, p < 0.001; Fig. 3A, D).

Following an injury, spontaneous activities can lead to DSFs in membrane potential [37]. We assessed these DSFs using an automated analysis similar to the method reported previously [30]. Our findings showed significantly increased DSF amplitudes when treated with 0.1 μM or 1 μM AEA in the Naive group (Naive: 2.19 ± 0.10 mV, n = 21; Naive + 0.1 μM AEA: 3.20 ± 0.19 mV, n = 19, p = 0.001; Naive + 1 μM AEA: 3.70 ± 0.23 mV, n = 23, p < 0.001; Fig. 3E, F). Large DSFs, defined by amplitudes > 5 mV, can be elicited occasionally [30]. In the naive group, large DSFs was observed in only 3 out of 21 DRG neurons (14.3%; Fig. 3G). However, administration of 1 μM AEA significantly increased the incidence of large DSFs to 47.8% (11 out of 23 neurons, p = 0.028; Fig. 3G). No significant changes were observed in the incidence of large DSFs in the presence of 0.01 μM or 0.1 μM AEA (Fig. 3G). In addition, rheobase was decreased in the group treated with 1 μM AEA compared to the naive group (Naive: 106.4 ± 8.0 pA, n = 47; Naive + 1 μM AEA: 70.5 ± 7.5 pA, n = 19, p = 0.029; Fig. 3H).

However, AEA did not exacerbate these hyperexcitable effects at any of the tested concentrations after SCI, possibly due to a ceiling effect. This includes the probability of sAP, the sAP frequency, RMP, DSF amplitudes, the incidence of large DSFs, and rheobase (Fig. 3). These results imply that the AEA concentration-dependent increase in neuronal excitability observed in the naive group is similar to the high neuronal excitability observed after SCI.

### AEA application after SCI promotes TRPV1-dependent transformation of AP

**Fig. 4:**
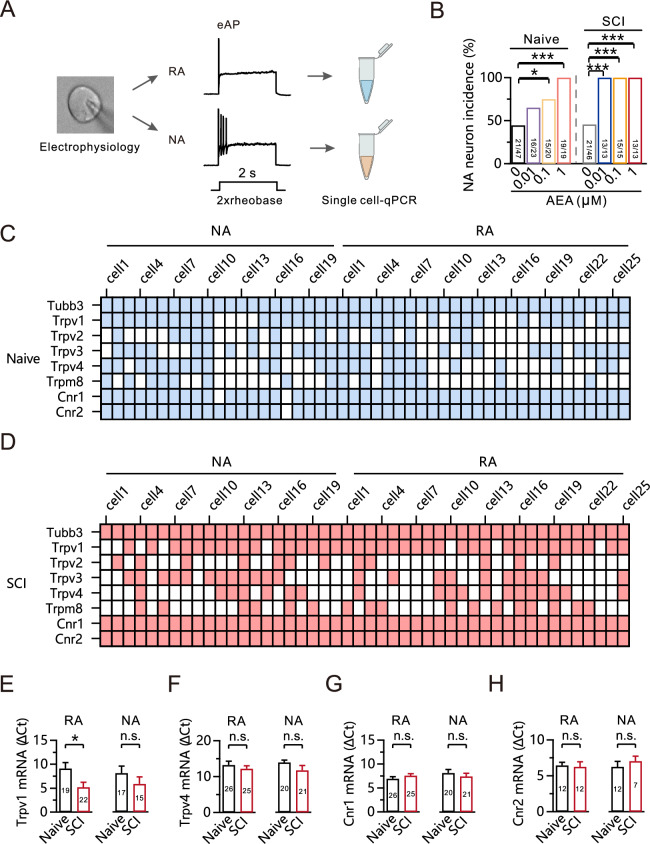
AEA application after SCI promotes TRPV1-dependent state transformation from RA to NA. **A** Schematic of the experimental design used for single-cell RT-qPCR after electrophysiological recordings. **B** Statistics for the NA neuron incidence using a Fisher’s exact test. *p < 0.05. ***p < 0.001. **C**, **D** The mRNA expression of the TRP family, *Cnr1*, and *Cnr2* in individual retrogradely traced DRG neurons in the naive (**C**) and 28 days post-SCI groups (**D**). Colored, expression. Colorless, no expression. **E** The relative mRNA expression level of Trpv1 in the RA and NA type neurons using a Mann Whitney test. *p < 0.05. n.s., not significant. **F** The relative mRNA expression level of Trpv4 in the RA (unpaired t-test) and NA (Mann Whitney test) type neurons. n.s., not significant. **G**–**H** The relative mRNA expression level of Cnr1 (**G**) and Cnr2 (**H**) in the RA and NA type neurons using an unpaired t-test. n.s., not significant

Nociceptors were classified in vitro as either RA or NA types based on their AP responses to a depolarizing current at twice the rheobase, a measure of induced neuronal excitability (Fig. 4A) [30]. External stimuli can induce transitions between these states [31]. Application of 1 μM or 0.1 μM AEA promoted the transition from RA to NA type and increased the occurrence of NA neurons compared to the naive group (Naive: 44.7%, 21 out of 47 neurons; Naive + 0.1 μM AEA: 75.0%, 15 out of 20 neurons, p = 0.032; Naive + 1 μM AEA: 100.0%, 19 out of 19 neurons, p < 0.001; Fig. 4B). However, this transition was eliminated by the presence of 0.01 μM AEA. Interestingly, we found that, though the AEA response to spontaneous neuronal activity was not further enhanced after SCI, AEA-induced transitions from RA to NA type were triggered by pre-treatment with lower AEA concentrations in the SCI group (SCI: 45.7%, 21 out of 46 neurons; SCI + 0.01 μM AEA: 100.0%, 13 out of 13 neurons, p < 0.001; SCI + 0.1 μM AEA: 100.0%, 15 out of 15 neurons, p < 0.001; SCI + 1 μM AEA: 100.0%, 13 out of 13 neurons, p < 0.001; Fig. 4B). This suggests an increased sensitivity of DRG neurons to AEA in inducing the transition from an RA to NA state following the injury.

To investigate the factors associated with the increased sensitivity of AEA post-SCI, we measured mRNA expression of the TRP family, *Cnr1*, and *Cnr2* in these neurons by single-cell qPCR following the determination of electrophysiological properties. We found that the TRP family, *Cnr1*, and *Cnr2* were expressed in all DRG neurons examined in both the naive and SCI groups. The transcript expression patterns fell into three categories: high expression (*Trpv1*: Naive, 76.6%, SCI, 80.4%; *Cnr1*: Naive, 95.7%, SCI, 100.0%; *Cnr2*: Naive, 97.9%, SCI, 100.0%), moderate expression (*Trpv3*: Naive, 63.8%, SCI, 47.8%; *Trpv4*: Naive, 51.1%, SCI, 41.3%), or low expression (*Trpv2*: Naive, 29.8%, SCI, 26.1%; *Trpm8*: Naive, 34.0%, SCI, 37.0%) (Fig. 4C, D).

We further analyzed the expression of these genes at the transcriptional level in NA and RA neurons. SCI led to an increase in *Trpv1* transcription in RA neurons, as the relative mRNA level was significantly increased compared to the naive group (Naive: 9.1 ± 1.2, n = 19; SCI: 5.2 ± 1.0, n = 22; p = 0.028; Fig. 4E). However, no significant differences were observed in *Trpv1* transcription levels in NA neurons between the naive and SCI groups (Fig. 4E). Furthermore, we found no significant differences in the transcription levels of *Trpv4* (Fig. 4F), *Cnr1* (Fig. 4G), and *Cnr2* (Fig. 4H) in both NA and RA neurons between the naive and SCI groups. These findings suggest that TRPV1 may play a key role in increasing the sensitivity of DRG neurons to AEA in the RA-NA state transformation following SCI.

### AEA induces hyperactivity in nociceptors via activation of TRPV1

**Fig. 5:**
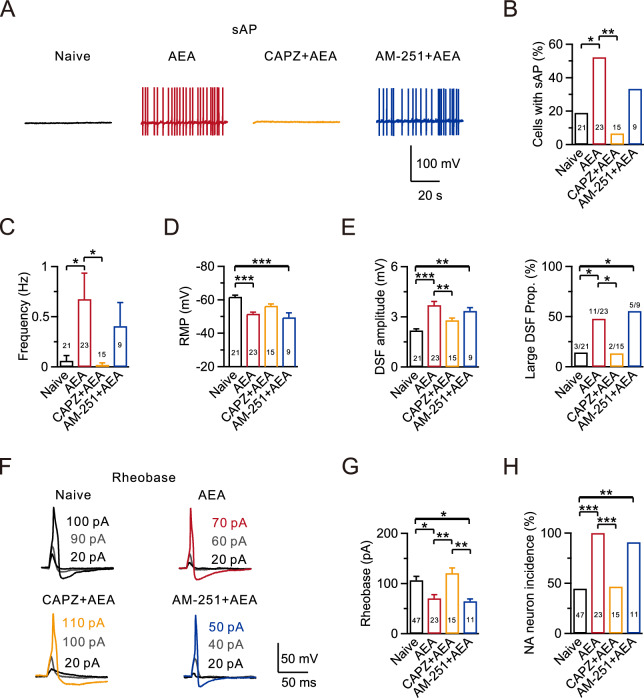
AEA induces hyperactivity in nociceptors via activation of TRPV1 receptor. **A** Representative sAP recordings using a whole-cell current-clamp configuration in the naive (black), AEA treatment (AEA, red), AEA treatment in the presence of CAPZ (CAPZ + AEA, yellow), and AEA treatment in the presence of AM-251 (AM-251 + AEA, blue) groups. **B**–**D** Statistics for the sAP probability, firing frequency, and RMP from various treatment groups (Naive, n = 21; AEA, n = 23; CAPZ + AEA, n = 15; AM-251 + AEA, n = 9). Comparisons among all four groups were performed using a Fisher’s exact test in B and a Kruskal–Wallis one-way ANOVA with Dunn’s multiple comparison tests in C and D. *p < 0.05, **p < 0.01, ***p < 0.001. **E** Statistics for the DSF amplitudes (left) and the incidence of large DSFs (right) in all four groups (Naive, n = 21; AEA, n = 23; CAPZ + AEA, n = 15; AM-251 + AEA, n = 9). Comparisons were made using a one-way ANOVA with Dunnett’s post hoc test on the left and a Fisher’s exact test on the right. *p < 0.05, **p < 0.01, ***p < 0.001. **F** Representative eAPs and their rheobase traces induced by depolarizing current injections in different groups (Naive, n = 47, black; AEA, n = 23, red; CAPZ + AEA, n = 15, yellow; AM-251 + AEA, n = 11, blue). **G** Statistics for the rheobase from (F) using a Kruskal–Wallis one-way ANOVA with Dunn’s multiple comparison test. *p < 0.05, **p < 0.01. **H** Statistics for the NA neuron incidence using a Fisher’s exact test. *p < 0.05, **p < 0.01, ***p < 0.001. Detailed statistical information is provided in Table 2

Having shown that AEA can potentiate neuronal activity after SCI, we further explored the downstream pathway involved in the induction of hyperactivity. We applied the TRPV1 antagonist (capsazepine, CAPZ, 10 μM) or CB1R antagonist (AM-251, 1 μM) to naive DRG neurons 1 h before the electrophysiological recording. Subsequently, DRG neurons isolated from naive mice were exposed to 1 μM AEA for 30 min. We found that the reversal of AEA-induced hyperactivity was linked to pre-treatment with CAPZ, not AM-251. This includes changes in the sAP probability and frequency, RMP, DSF amplitude, incidence of large DSFs, rheobase, and the incidence of an NA state (Fig. 5A–F, Table 2). These findings suggest that SCI-induced hyperexcitability primarily stems from activation of TRPV1 by increased AEA levels.

**Table 2:** Electrophysiological properties in neurons from treated and untreated groups

Property	Naive	AEA	CAPZ + AEA	AM251 + AEA	Test
Cells with sAP (%)	19.0	52.2	6.7	33.3	Fisher’s exact test: Naive vs. AEA, p = 0.031; AEA vs. CAPZ + AEA, p = 0.005
Frequency (Hz)	0.06 ± 0.05	0.68 ± 0.26	0.02 ± 0.02	0.41 ± 0.24	K–W test: Naive vs. AEA, p = 0.049; AEA vs. CAPZ + AEA, p = 0.013
RMP (mV)	− 61.8 ± 1.0	− 51.6 ± 1.0	− 56.4 ± 1.0	− 49.4 ± 2.7	K–W test: Naive vs. AEA, p < 0.001; Naive vs. AM251 + AEA, p < 0.001
DSF amplitude (mV)	2.19 ± 0.10	3.70 ± 0.23	2.79 ± 0.13	3.35 ± 0.19	One-way ANOVA: Naive vs. AEA, p < 0.001; Naive vs. AM251 + AEA, p = 0.001; AEA vs. CAPZ + AEA, p = 0.003
Large DSF (%)	14.3	47.8	13.3	55.6	Fisher’s exact test: Naive vs. AEA, p = 0.025; Naive vs. AM251 + AEA, p = 0.032; AEA vs. CAPZ + AEA, p = 0.039
Rheobase (pA)	106.4 ± 8.0	70.5 ± 7.5	120.7 ± 10.4	64.6 ± 4.6	K–W test: Naive vs. AEA, p = 0.041; Naive vs. AM251 + AEA, p = 0.022; AEA vs. CAPZ + AEA, p = 0.006; Naive vs. CAPZ + AEA, p = 0.003
NA neuron incidence (%)	44.7	100.0	46.7	90.9	Fisher’s exact test: Naive vs. AEA, p < 0.001; Naive vs. AM251 + AEA, p < 0.001; AEA vs. CAPZ + AEA, p = 0.007

Each value is the mean ± SEM or incidence (%). K–W test, Kruskal–Wallis one-way ANOVA with Dunn’s multiple comparison test; One-way ANOVA, one-way ANOVA with Dunnett’s post hoc test

AEA, anandamide; CAPZ, capsazepine; AP, action potential; RMP, resting membrane potential; DSF, depolarizing spontaneous fluctuation; NA, non-accommodating

### TRPV1 inhibitor or CB1R agonist eliminates SCI-induced hyperexcitability

**Fig. 6:**
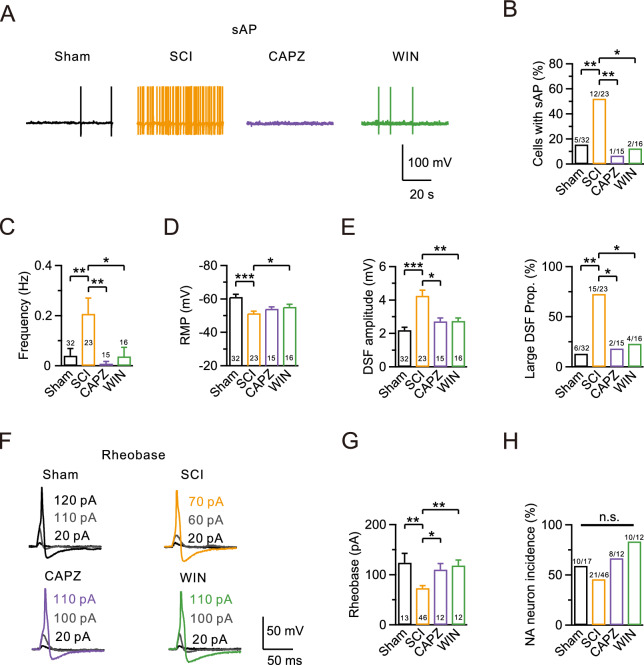
TRPV1 inhibitor or CB1R agonist eliminates SCI-induced hyperexcitability. **A** Representative sAP recordings using a whole-cell current-clamp configuration in the sham (black), SCI (yellow), CAPZ-treated SCI (10 μM, purple), and WIN-treated SCI (1 μM, green) groups. **B**–**D** Statistics for the sAP probability, firing frequency, and RMP from various treatment groups (Sham, n = 32; SCI, n = 23; CAPZ, n = 15; WIN, n = 16). Comparisons among all four groups were performed using a Fisher’s exact test in B, a Kruskal–Wallis one-way ANOVA with Dunn’s multiple comparison test in C, and a one-way ANOVA with Dunnett’s post hoc test in D. *p < 0.05. **p < 0.01. ***p < 0.001. **E** Statistics for the DSF amplitude (left) and the incidence of large DSFs (right) in all four groups (Sham, n = 32; SCI, n = 23; CAPZ, n = 15; WIN, n = 16). Comparisons were carried out using a Kruskal–Wallis one-way ANOVA with Dunn’s multiple comparison test on the left and a Fisher’s exact test on the right. *p < 0.05, **p < 0.01, ***p < 0.001. **F** Representative eAPs and their rheobase traces induced by depolarizing current injections in different groups (Sham, n = 13, black; SCI, n = 46, yellow; CAPZ, n = 12, purple; WIN, n = 12, green). **G** Statistics for the rheobase from (F) using a Kruskal–Wallis one-way ANOVA with Dunn’s multiple comparison test. *p < 0.05, **p < 0.01. **H** Statistics for the NA neuron incidence using a Fisher’s exact test. n.s., not significant. Detailed statistical information is provided in Table 3

Our results indicate that AEA enhances neuronal excitability after SCI via activated TRPV1, but the significance of elevated CB1R levels after SCI remains unclear. Therefore, we applied CAPZ (10 μM) or the exogenous cannabinoid agonist WIN55212-2 (WIN, 1 μM) [38] for 1 h to inhibit TRPV1 or activate CB1R in DRG neurons 28 days post-SCI. We then assessed the neurophysiological activity via whole-cell recordings. Administration of either CAPZ or WIN reduced SCI-induced hyperexcitability, including the sAP probability and frequency, RMP, DSF amplitude, incidence of large DSFs, and rheobase. However, the proportion of NA neurons remained unchanged in the absence of AEA (Fig. 6A–F, Table 3). In summary, inhibiting TRPV1 or activating CB1R can reduce the SCI-induced hyperexcitability of DRG neurons in vitro.

**Table 3:** Electrophysiological properties in neurons from sham and SCI groups

Property	Sham	SCI	SCI + CAPZ	SCI + WIN55212-2	Test
Cells with sAP (%)	15.6	52.2	6.7	12.5	Fisher’s exact test: Sham vs. SCI, p = 0.007; SCI vs. CAPZ, p = 0.005; SCI vs. WIN, p = 0.017
Frequency (Hz)	0.04 ± 0.03	0.21 ± 0.06	0.01 ± 0.01	0.04 ± 0.04	K–W test: Sham vs. SCI, p = 0.006; SCI vs. CAPZ, p = 0.006; SCI vs. WIN, p = 0.021
RMP (mV)	− 60.2 ± 1.5	− 51.4 ± 1.3	− 54.1 ± 1.2	− 55.2 ± 1.5	One-way ANOVA: Sham vs. SCI, p < 0.001; Sham vs. SCI, p = 0.036
DSF amplitude (mV)	2.36 ± 0.19	4.00 ± 0.20	2.71 ± 0.15	2.67 ± 0.16	K–W test: Sham vs. SCI, p < 0.001; SCI vs. CAPZ, p = 0.012; SCI vs. WIN, p = 0.004
Large DSF (%)	18.8	65.2	13.3	25.0	Fisher’s exact test: Sham vs. SCI, p = 0.001; SCI vs. CAPZ, p = 0.038; SCI vs. WIN, p = 0.030
Rheobase (pA)	123.8 ± 18.5	73.0 ± 5.1	110.0 ± 12.2	118.3 ± 11.1	K–W test: Sham vs. SCI, p = 0.002; SCI vs. CAPZ, p = 0.043; SCI vs. WIN, p = 0.008
NA neuron incidence (%)	58.8	45.7	66.7	83.3	Fisher’s exact test: n.s

Each value is the mean ± SEM or incidence (%). K–W test, Kruskal–Wallis one-way ANOVA with Dunn’s multiple comparison test; One-way ANOVA, one-way ANOVA with Dunnett’s post hoc test

SCI, spinal cord injury; CAPZ, capsazepine; AP, action potential; RMP, resting membrane potential; DSF, depolarizing spontaneous fluctuation; NA, non-accommodating

### Increased sensitivity of AEA-TRPV1 increases cytosolic calcium after SCI

**Fig. 7:**
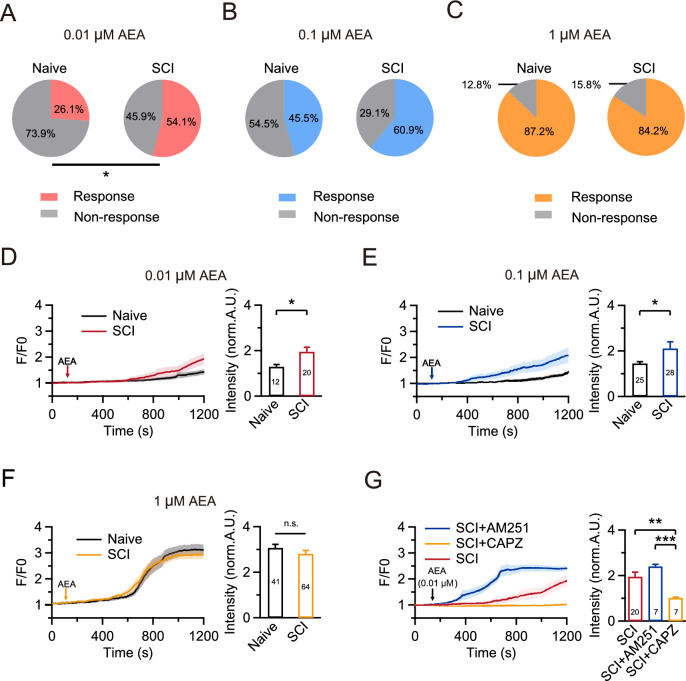
The increased sensitivity of AEA-TRPV1 increases cytosolic calcium after SCI. **A** Pie charts showing the proportion of responding and non-responding neurons to 0.01 μM AEA using calcium imaging in both naive and SCI groups. Comparisons between groups were carried out using a Fisher’s exact test. *p < 0.05. **B** Similar to (A), but in the presence of 0.1 μM AEA. n.s., not significant. **C** Similar to (A), but in the presence of 1 μM AEA. n.s., not significant. **D** Left, neuronal [Ca^2+^]_cyto_ responses to 0.01 μM AEA in both naive and SCI groups. AEA was administered 120 s after the start of the recording. Right, statistics for the normalized fluorescence intensity (normalized to the first 120 s) of [Ca^2+^]_cyto_ in neurons in the naive and SCI groups using a Mann Whitney test. *p < 0.05. **E** Similar to (**D**), but in the presence of 0.1 μM AEA. *p < 0.05. **F** Similar to (D), but in the presence of 1 μM AEA. n.s., not significant. **G** AEA (0.01 μM) induced changes in [Ca^2+^]_cyto_ on DRG neurons after SCI with pre-treatment with CAPZ (10 μM, yellow) or AM-251 (1 μM, blue)

AEA increases cytosolic Ca^2+^ by activating the non-selective cation channel TRPV1, leading to the potentiation of pain-related neurotransmission. Conversely, CB1R activation can inhibit calcium influx by modulating presynaptic VGCCs [14]. To investigate the effects of AEA on cytosolic calcium after SCI, we used calcium imaging to measure the [Ca^2+^]_cyto_ before and after AEA administration at various concentrations in both the naive and SCI groups. After obtaining a baseline [Ca^2+^]_cyto_ for 120 s as a control, AEA was applied to DRG neurons (≤ 30 μm) in vitro. In the naive group, 12 out of 46 neurons (26.1%) showed responses to 0.01 μM AEA, whereas 20 out of 37 neurons (54.1%) responded to the same concentration 28 days post-SCI (p = 0.002; Fig. 7A). At higher AEA concentrations (0.1 μM or 1 μM), we found no significant difference in the proportion of responding neurons (Fig. 7B, C). Subsequently, we found that responding neurons in the naive and SCI groups had different levels of increase in [Ca^2+^]_cyto_ after AEA administration. Approximately 1200 s after administering 0.01 μM AEA, the [Ca^2+^]_cyto_ of responding neurons in the naive group increased to 129.5 ± 9.9% compared to baseline (Fig. 7D). We observed a higher increase in [Ca^2+^]_cyto_ in response to 0.01 μM AEA 28 days post-SCI compared to the naive group (194.8 ± 20.2%; p = 0.018; Fig. 7D). Similarly, there was a higher increase in [Ca^2+^]_cyto_ in response to 0.1 μM AEA 28 days post-SCI compared to the naive group (Naive: 144.8 ± 7.6%; SCI: 210.5 ± 29.0%; p = 0.042; Fig. 7E). However, no significant changes were observed in the presence of 1 μM AEA (Fig. 7F). These findings suggest that AEA induces more calcium influx into the DRG neurons after SCI than in the naive group.

As to why AEA induces a greater [Ca^2+^]_cyto_ increase following SCI than in the naive group, we propose two hypotheses. First, despite the enduring inhibitory effect of CB1R on calcium influx post-SCI, the overall impact of AEA may still lean towards increasing the calcium influx due to TRPV1 upregulation. Second, the function of CB1R may shift to promote intracellular calcium influx after SCI. To explore the factors leading to changes in the effects of AEA on cytosolic calcium after SCI, we applied CAPZ (10 μM) or AM-251 (1 μM) to neurons 28 days post-SCI starting 30 min prior to intracellular calcium imaging. Subsequently, we administered 0.01 μM AEA to neurons after obtaining a baseline [Ca^2+^]_cyto_ for 120 s as a control. Although pre-treatment with AM-251 did not further increase the peak value of [Ca^2+^]_cyto_, it affected the timing of the response. Compared to the group without pre-treatment, we observed a faster increase in [Ca^2+^]_cyto_ following administration of 0.01 μM AEA in the AM-251 pre-treatment group (Fig. 7G). This suggests an inhibitory effect of CB1R on calcium influx post-SCI. Furthermore, we did not observe a significant increase in [Ca^2+^]_cyto_ after administering 0.01 μM AEA with CAPZ pre-treatment (Fig. 7G), implying an increase in [Ca^2+^]_cyto_ due to TRPV1 activation.

## Discussion

**Fig. 8:**
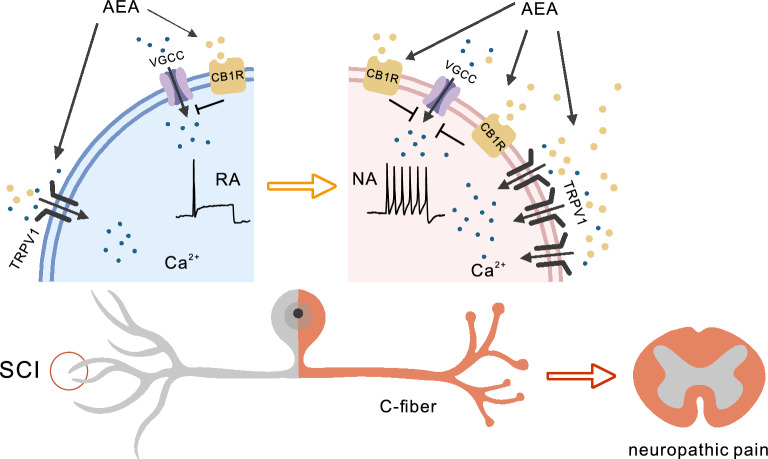
Schematic of the pathological mechanism of AEA-TRPV1/CB1R-induced hyperexcitability in nociceptors post-SCI. Increased AEA content induces transformation from the RA to NA state and hyperexcitability in nociceptors via the AEA-TRPV1-Ca^2+^ pathway after SCI. The early increase in CB1R inhibits VGCCs to maintain [Ca^2+^]_cyto_ at low levels, suggesting adaptive neuroprotection via the AEA-CB1R signaling pathway. However, increasing levels of AEA and TRPV1 post-SCI leads to nociceptor maladaptation, inducing an increase in [Ca^2+^]_cyto_ and transforming the RA to NA state, which contributes to chronic neuropathic pain. VGCC, voltage-gated calcium channel; TRPV1, transient receptor potential vanilloid 1; AEA, anandamide; CB1R, cannabinoid type-1 receptor

Chronic peripheral sensitization of nociceptors drives neural pathways that result in sustained pain after SCI [39, 40]. In the present study, we found that an increase in AEA content after SCI leads to hyperexcitability in nociceptors via the AEA-TRPV1 signaling pathway. We also observed an early-stage increase in the neuroprotective receptor CB1R, suggesting a potential adaptive neuroprotective role for AEA-CB1R signaling shortly after SCI. However, due to the increase in both AEA and TRPV1, nociceptors transition to a maladaptive state marked by hyperexcitability and hypersensitivity, which contributes to the development of chronic neuropathic pain post-SCI (Fig. 8).

### Pathological alterations in AEA-TRPV1 lead to hyperexcitability of nociceptors after SCI

How AEA is altered at the spinal level under pathological conditions, such as neuropathic pain, is still controversial [41]. Previous studies suggested that AEA levels increase in the spinal cord and DRGs after peripheral nerve injuries [16, 42, 43]. However, Kinsey et al. indicated no significant change in AEA levels in the spinal cord after such an injury [44]. The international SCI pain classification divides neuropathic pain post-SCI into “at-level” and “below-level” pain [45]. We observed a significant increase in AEA content in DRGs and the spine below the injury level 28 days post-SCI (Fig. 1), indicating activation of the endocannabinoid system as a compensatory mechanism. This activation may reduce neuronal excitability and modulates pain and inflammation [14]. Furthermore, disruption of the endocannabinoid system homeostasis by activated glial cells after SCI may contribute to this increase [46].

Previous studies investigated AEA changes after SCI and found no significant difference in AEA in the epicenter and rostral region between 28 days post-SCI and the sham group [36]. However, AEA increased in the early stages of the injury [35]. The varying levels of AEA in different spinal levels could be due to the different characteristics and pathological mechanisms of these pain types. Supporting this, [5] found that sAP incidence did not increase in DRG neurons above the T10 contusion site, but it significantly increased in DRG neurons below the contusion site. We also observed that the timing of the sAP increase in nociceptors is consistent with the increase in AEA content below the injury level (Fig. 1). This implies that SCI-induced hyperexcitability in nociceptors is related to the increased AEA content.

Recent studies indicate that an increase in excitatory or sensitizing signals, or a decrease in inhibitory signals, may contribute to persistent nociceptor hyperactivity [37]. However, some external signals modulate pain-related hyperactivity via a variety of mechanisms. For example, SCI induces an increase in the release of macrophage migration inhibitory factor (MIF) in nociceptors. Though low MIF levels excite nociceptors, higher levels induce a hypoexcitable state [31]. AEA is generally considered an inhibitory chemical signal due to its ability to enhance CB1R-mediated pain relief in central nerves, but our study found that increased AEA in DRGs after SCI may pathologically shift the state from anti-nociceptive to pro-nociceptive. One possible mechanism for this pathological change could be the concentration of AEA. We demonstrated that the administration of 1 μM AEA can mimic the pathological behavior of nociceptors after SCI, whereas a low dose of AEA had no effect (Fig. 3). Our findings are in line with previous studies suggesting that low concentrations of AEA primarily exert a CB1R-mediated anti-nociception effect, whereas high concentrations of AEA stimulate TRPV1, exciting nociceptors and causing the release of neuropeptides into the dorsal spinal cord, producing the nociceptive effect [19, 47]. Another potential mechanism could be changes in the inherent sensitivity that promote nociceptor hyperactivity [10]. When the balance between TRPV1 and CB1R is disrupted following injury, the sensitivity to external chemical signals, including AEA, increases, leading to excitation.

The extracellular environment can change both the intrinsic sensitivity and expression of receptors. For example, under neuroinflammatory conditions following SCI, such as in the presence of bradykinin or prostaglandins, the sensitivity of TRPV1 to AEA is increased [48]. Our study observed a parallel increase in AEA and TRPV1 in DRG after SCI (Fig. 2). S Hong et al. also found that exposing naive DRG neurons to high concentrations of AEA in vitro enhances TRPV1 expression [18]. This led us to hypothesize that the increase in TRPV1 post-SCI may be due to the increased AEA levels. However, whether externally inhibiting AEA production can inhibit the upregulation of TRPV1, thus potentially reducing pain, remains unclear.

### Adaptive changes in CB1R may serve as a protective response to inhibit SCI-induced hyperexcitability

When exposed to nociceptive stimuli, protective receptors undergo adaptive changes [49]. Our study found an early increase in CB1R after SCI. This suggests that the increased expression of CB1R in both CGRP- and IB4-positive neurons, which are associated with C-fibers, could act as a protective response to inhibit SCI-induced hyperexcitability. Interestingly, an increase in CB1R was also noticed in large DRG neurons identified by NF200, a non-nociceptive mechanoreceptor marker [50]. Although the impact of and changes in CB1R on primary receptors after SCI are not fully understood, several studies have investigated its function in the brain and spinal cord. An increase in CB1R during the early stages of SCI has been suggested to assist in motor function recovery following incomplete SCI [35]. Other studies suggest that interactions among CB1R, C–C chemokines, and TRPV1 may contribute to SCI-induced brain alterations, leading to emotional-affective pain responses and the development of central pain after SCI [51].

### Electrophysiological state transitions and hyperexcitability mechanisms post-SCI

Odem et al. [30] identified two electrophysiological states of DRG neurons under stimulated conditions, termed the NA and RA states. Following SCI, the increase in sAP is driven primarily by NA neurons. In vitro, NA neurons made up 69% of the population, whereas RA neurons made up 31%, a ratio similar to our findings. The NA-RA state changes with the external signal [31], suggesting that NA and RA represent distinct functional states, not fixed phenotypes. The RA-NA transformation represents a shift in neuronal adaptability to sustained stimuli, with RA neurons maintaining a hypoexcitable state and NA neurons exhibiting repetitive firing in response to prolonged stimuli [30]. Clinically, these mechanisms may manifest as distinct symptoms. sAPs are closely linked to spontaneous and persistent pain, while the RA-NA transformation may explain contrasting symptoms such as hypoalgesia (RA neurons) and hyperalgesia/allodynia (NA neurons) [5, 30]. Notably, the RA-NA state transformation could represent a broader adaptation mechanism influencing neuronal behavior beyond nociceptive pathways. Our study linked these electrophysiologically defined neuron types to molecular markers using single-cell PCR. We found that the potential for transition between NA and RA types is related to the expression of TRPV1 mRNA. This correlation clarifies the increased sensitivity to AEA in inducing TRPV1-dependent transition from the RA to NA state following injury. Understanding these connections could provide valuable insights into targeting TRPV1 or modifying the RA-NA balance as therapeutic strategies for neuropathic pain management.

We also found that AEA can dose-dependently and persistently increase DSFs, mimicking the random fluctuations in RMP observed post-SCI (Fig. 3). This heightened excitability can be reduced by inhibiting TRPV1 or activating CB1 receptors (Figs. 5 and 6). Previous studies identified DSFs as transient components of RMP, associated with the irregular firing patterns of nociceptor sAP [30]. Under the hyperactive conditions of neuropathic pain, the increase in DSFs contributes to spontaneous activity [52]. However, the biophysical and cellular signaling mechanisms underlying the generation and amplification of DSFs remain unclear. Our findings provide additional insights into the mechanisms of DSFs, which could help create more precise treatments for spontaneous pain.

### Regulation of cytosolic calcium by CB1R and TRPV1 in neuropathic pain after SCI

Ca2⁺ plays a crucial role in maintaining the normal function of the nervous system and regulating its dynamic changes [53]. Our study suggests that CB1R and TRPV1 can regulate cytosolic calcium levels after SCI, which has also been shown in many other cases. For example, nerve growth factor (NGF) levels increase during inflammation, injury, or chronic pain states [54, 55]. Applying high levels of NGF in vitro increases the proportion of intracellular calcium flow in DRG neurons after AEA activates TRPV1. In addition, in the presence of high NGF, crosstalk between receptors CB1R and TRPV1 is enhanced, further increasing calcium inflow induced by the AEA-TRPV1 pathway [56]. However, we found that CB1R agonists can still inhibit the excitability of DRG neurons after SCI, suggesting that the upregulated CB1R following SCI can continue to exert inhibitory effects. This finding suggests a promising analgesic strategy using the combination of CB1R agonists and TRPV1 inhibitors for neuropathic pain after SCI.

## Conclusions

In conclusion, increased AEA content post-SCI induces nociceptor hyperexcitability via the AEA-TRPV1 pathway. An early increase in CB1R suggests adaptive neuroprotection by AEA-CB1R signaling. However, increasing levels of AEA and TRPV1 lead to nociceptor maladaptation, contributing to chronic neuropathic pain post-SCI. This evidence suggests that endocannabinoids and their receptors are altered in a specific manner in the pathological state of SCI, which may support a more targeted approach to the development of cannabinoid-based pain medications.

## Supplementary Information

Additional file 1.

## Data Availability

Data are available upon reasonable request. Please contact the corresponding author.

## Abbreviations

ATP: Adenosine triphosphate
AEA: Anandamide (N-arachidonoyl-ethanolamine)
BMS: Basso Mouse Scale
BCA: Bicinchoninic acid
CGRP: Calcitonin gene-related peptide
CAPZ: Capsazepine
DSFs: Depolarizing spontaneous fluctuations
ECL: Enhanced chemiluminescence
eAP: Evoked action potentials
HRP: Horseradish peroxidase
IB4: Isolectin B4
LPS: Lipopolysaccharide
MIF: Macrophage migration inhibitory factor
NGF: Nerve growth factor
NF200: Neurofilament-200
PFA: Paraformaldehyde
PBS: Phosphate buffered saline
PVDF: Polyvinylidene fluoride
ROIs: Regions of interest
SNL: Spinal nerve ligation
sAPs: Spontaneous action potentials
VGCCs: Voltage-gated calcium channels
WIN: WIN55212-2
