# Supplementary material for: Maladaptive changes in the homeostasis of AEA-TRPV1/CB1R induces pain-related hyperactivity of nociceptors after spinal cord injury
Source: Cell Biosci. 2025 Jan 9;15:2. doi: 10.1186/s13578-025-01345-6 (PMC11720958; doi:10.1186/s13578-025-01345-6)
Supplement: Supplementary file 1 — Additional file 1. [file 13578_2025_1345_MOESM1_ESM.docx]

**Supplementary Information**

**I. TRPV1 and CB1R are upregulated in nociceptors following SCI**


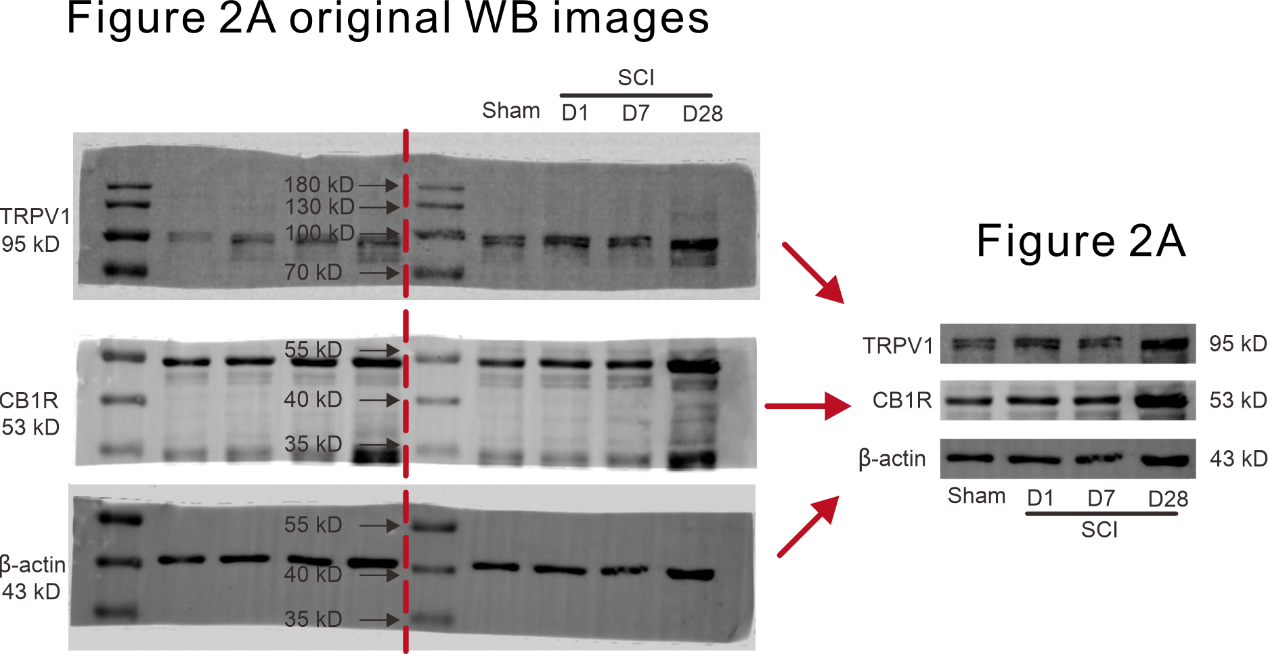


Figure S1. Original uncropped blot images of Figure 2A showing of TRPV1 and CB1R protein levels in DRGs from the sham group and at different time points after SCI.
